# Editorial: Prescribing psychotropics: Misuse, abuse, dependence, withdrawal and addiction, Volume II

**DOI:** 10.3389/fpsyt.2022.1053896

**Published:** 2022-10-18

**Authors:** Stefania Chiappini, Fabrizio Schifano, Giovanni Martinotti

**Affiliations:** ^1^Psychopharmacology, Drug Misuse and Novel Psychoactive Substances Research Unit, School of Life and Medical Sciences, University of Hertfordshire, Hatfield, United Kingdom; ^2^Department of Neuroscience, Imaging and Clinical Sciences, “G. D'Annunzio” University, Chieti, Italy

**Keywords:** drug abuse, drug misuse, prescription drug misuse, pharming, drug diversion, addiction, pharmacovigilance

In recent decades, the landscape of drug abuse has changed due to the emergence in the drug scene of molecules known as new psychoactive substances (NPS) (1–3). In addition, the phenomenon of recreational use of pharmaceuticals which have not yet been controlled because theoretically considered to have no diversion potential, but have shown potential abuse liability, acute drug toxicity presentations and psychiatric consequences (4–10), has emerged. Several molecules, including opioids, benzodiazepines, and other central nervous system (CNS) depressants (including sedatives and hypnotics) and prescription stimulants, e.g., amphetamines, have been reported to be abused and diverted (11–13). Furthermore, several over-the-counter (OTC) drugs, including loperamide, dextromethorphan, promethazine and benzydamine, have been increasingly reported to be abused by young adult populations in the context of pharming and pharm-parties (4, 8, 9, 11, 14–16). The use of drugs would be facilitated by the perception that these molecules are socially more acceptable/less stigmatizing and also safer than other illicit substances, and by the lack of their detection in standard drug controls; moreover, they can be extremely easy to obtain online *via* the web, avoiding the risk of legal problems associated with illicit drug purchases; finally, the possibility of interactions between prescription drugs and other licit/illicit substances, emphasizing drug-related consequences due to interactions with NPS, make them more attractive (8, 9, 11, 14–16). In this context, the Corona Virus Disease (COVID)-19 pandemic, having an impact on drug markets, through the reduction and the disappearance of many types of drugs at street level and price increases, may have led to a further increase in risks for drug users, for example by increasing the variability of drug purity through adulteration with other molecules unknown to the user. This clearly may have encouraged a shift to riskier behaviors, including the consumption of available and potent drugs such as street benzodiazepines and synthetic opioids, if access to those previously used becomes limited; led to changes in drug use levels; or finally caused a relapse, if drug diversion/addiction had already been treated (17–22). Finally, the use of highly psychoactive molecules including designer benzodiazepines, new synthetic opioids (NSO), and illicit fentanyl analogs has become widespread worldwide, posing an upcoming global public health challenge due to the high abuse potential and severe adverse effects, including fatalities (23–25). For instance, the National Institute on Drug Abuse (NIDA) reported that in 2020, 16% of opioid overdose deaths also involved benzodiazepines, the latter being found in the illicit opioid supply in some areas, which could mean that people were taking benzodiazepines in combination with illicit opioids knowingly or unknowingly (26). Similarly, through analysis of death certificates, fentanyl was found to be involved in 90% of all US opioid-related deaths along with methamphetamine and cocaine (27, 28). Medications may be added to illicit drug supply for many reasons, but sometimes the medications produce their own toxicity; in fact, pharmacologically active compounds, impurities, or breakdown products from drug manufacturing and industrial chemicals, collectively referred to as “toxic adulterants,” are now found in street drugs. These include, but are not limited to: antipsychotics, antidepressants, anxiolytics, antihistamines, anthelmintics, anesthetics, anti-inflammatories, antipyretics, analgesics, antispasmodics, antiarrhythmics, antimalarials, veterinary medications, bronchodilators, expectorants, sedatives, muscle relaxers, natural/synthetic hallucinogens, decongestants, NPS, industrial compounds, fungicides, etc. Unfortunately, routine clinical or workplace drug testing cannot detect all these toxic adulterants (29).

Consistent with these issues, this Research Topic has collected five contributions, including two original articles, two case reports, and a study protocol. In their study, Savulich et al. investigated the relationship between NPS/polydrug use and neuropsychological functioning measures through the Cambridge Neuropsychological Test Automated Battery (CANTAB) and the EMOTICOM test battery in a sample including adult male recreational users without psychiatric comorbidities (“psychonauts,” *n* = 27) and healthy control volunteers (*n* = 35). Overall, by distinguishing tasks according to “hot” cognitive processes, that are highly influenced by emotion, from “cold” cognitive processes, which are largely independent of emotional influence, they found that recreational NPS users exhibited elevated “hot” cognition, whereas “cold” cognitive dysfunction along with risk-taking behaviors were not pronounced, the latter protecting them from the negative consequences of chronic NPS/polydrug use. Concerning the growing alarm worldwide registered, Specka et al. investigated NSO-related overdoses and deaths in Germany in a sample of 252 opioid-dependent patients referred for in-patient detoxification treatment, recording a lifetime use of heroin and opioid analgesics respectively of 99.2 and 30.4%, while the reported lifetime of NSO/fentanyl use, their regular use and ingestion in the 30 days prior to admission were respectively 8.7, 1.6, and 0.8 %. Although the data may be reassuring in light of the current global opioid crisis, opioid use should be monitored as well as the time course of opioid use which has shown that heroin typically anticipates analgesic opioid use and NSO; in addition, NSO/fentanyl use might be sometimes under-recorded or underestimated because users may not be aware of the NSO they are about to ingest or the specific NSO may be mixed with other psychoactive substances, e.g., cocaine and heroin, or counterfeited, and may not be detected in common drug tests. Benzodiazepines are still among the most commonly prescribed psychotropic medications worldwide. The possibility that chronic use of benzodiazepines can cause deficits in learning, attention, memory, depression and occurrence of injurious falls, road traffic and other accidents, has been well known for a long time. The risk of dependence after long-term use has been largely described, as reflected in the appearance of a series of symptoms when the drug is abruptly withdrawn. The United Nations reports that the non-medical use of benzodiazepines is a well-known phenomenon and a growing public health problem during the pandemic, whose magnitude is difficult to estimate, mainly due to the lack of monitoring and data collection in most countries (30, 31). Indeed, patients who have started using benzodiazepines to treat anxiety and insomnia disorders might end up using them inappropriately, deliberately abusing them. In this case, benzodiazepines are taken in an attempt to fight anxiety or to increase the effect of other drugs such as alcohol or opioids, this being part of a pattern of polyaddiction. In the case report described by Li et al., a 46-year-old male with a 24-year history of schizophrenia overused clonazepam combined with clozapine for 10 years up to 26 and 1,275 mg day respectively, and was medically treated in hospital for 1 month in order to gradually reduce their daily dose to clonazepam 2 mg and clozapine 450 mg day, using therapeutic drug monitoring (daily dosage and plasmatic concentration) and pharmacogenetic testing. Similarly, Mao et al. described the case of a 25-year-old man misusing zolpidem at high dosage (up to 80 mg day, while normal daily dosage is 10 mg) for 2 years, performing abnormal and high-risk behaviors, such as sleep walking, sleep driving and sleep eating (32); after admission the patient underwent a typical benzodiazepine-withdrawal toxidrome characterized by rebound insomnia, anxiety, craving, paraesthesia, influenza-like symptoms, tonic-clonic-type seizures and hallucinations (33, 34). Consistent with the reported issues, Casari et al. proved the Verona Detox approach for detoxification from high dose of benzodiazepine and Z-drugs, by means of medical-assisted administration of flumazenil, a selective benzodiazepine receptor antagonist, is safe and effective.

In conclusion, the misuse and abuse of prescription/OTC drugs is a cause for major concern, especially in vulnerable individuals or in some contexts, such as polysubstance abuse, history of drug abuse or drug addiction. The use of concomitant substances or of high/supra-high doses for recreational purposes may cause unpredictable effects, resulting clinical toxidromes, overdoses or drug-related fatalities. The present situation represents a challenge for psychiatry, public health, and drug-control policies with enormous implications for clinical practice in terms of harm reduction strategies, preventable morbidity, and mortality. Controlling the problem of prescription and OTC drugs misuse and abuse is challenging, as it is necessary to achieve a high level of safety for consumers without restricting access to medications in general for those who continue to use them safely. In this regard, prevention and early education on substance abuse in vulnerable categories, such as young teens, are critical, but also other groups where problems have been observed.

## Author contributions

SC, FS, and GM contributed equally to this Editorial of the Research Topic on prescribing psychotropics' misuse that they edited. All authors contributed to the article and approved the submitted version.

## Conflict of interest

Author FS was a member of the UK Advisory Council on the Misuse of Drugs (ACMD; 2011-2019) and is currently a member of the EMA Advisory Board (Psychiatry). Author GM has been a consultant and/or a speaker and/or has received research grants from Angelini, Doc Generici, Janssen-Cilag, Lundbeck, Otsuka, Pfizer, Servier, and Recordati. The remaining author declares that the research was conducted in the absence of any commercial or financial relationships that could be construed as a potential conflict of interest.

## Publisher's note

All claims expressed in this article are solely those of the authors and do not necessarily represent those of their affiliated organizations, or those of the publisher, the editors and the reviewers. Any product that may be evaluated in this article, or claim that may be made by its manufacturer, is not guaranteed or endorsed by the publisher.
